# Harnessing Prebiotics to Improve Type 2 Diabetes Outcomes

**DOI:** 10.3390/nu16203447

**Published:** 2024-10-11

**Authors:** Oana C. Iatcu, Sevag Hamamah, Mihai Covasa

**Affiliations:** 1 Department of Biomedical Sciences, College of Medicine and Biological Science, University of Suceava, 720229 Suceava, Romania; oana.iatcu@usm.ro; 2 Department of Basic Medical Sciences, College of Osteopathic Medicine, Western University of Health Sciences, Pomona, CA 91766, USA; sevag.hamamah@westernu.edu; 3 Department of Internal Medicine, Scripps Mercy Hospital, San Diego, CA 92103, USA

**Keywords:** inulin, resistant starch, oligosaccharides, β-glucan, polyphenols

## Abstract

The gut microbiota, a complex ecosystem of microorganisms in the human gastrointestinal tract (GI), plays a crucial role in maintaining metabolic health and influencing disease susceptibility. Dysbiosis, or an imbalance in gut microbiota, has been linked to the development of type 2 diabetes mellitus (T2DM) through mechanisms such as reduced glucose tolerance and increased insulin resistance. A balanced gut microbiota, or eubiosis, is associated with improved glucose metabolism and insulin sensitivity, potentially reducing the risk of diabetes-related complications. Various strategies, including the use of prebiotics like inulin, fructooligosaccharides, galactooligosaccharides, resistant starch, pectic oligosaccharides, polyphenols, β-glucan, and *Dendrobium officinale* have been shown to improve gut microbial composition and support glycemic control in T2DM patients. These prebiotics can directly impact blood sugar levels while promoting the growth of beneficial bacteria, thus enhancing glycemic control. Studies have shown that T2DM patients often exhibit a decrease in beneficial butyrate-producing bacteria, like *Roseburia* and *Faecalibacterium*, and an increase in harmful bacteria, such as *Escherichia* and *Prevotella*. This review aims to explore the effects of different prebiotics on T2DM, their impact on gut microbiota composition, and the potential for personalized dietary interventions to optimize diabetes management and improve overall health outcomes.

## 1. Introduction

The gut microbiota is a diverse community of microorganisms residing in the human gastrointestinal (GI) tract. This complex ecosystem, composed of trillions of microbes—including bacteria, viruses, fungi, and other microorganisms—plays a significant role in influencing normal physiological processes and disease susceptibility through its metabolic activities and interactions with the host [1]. It is crucial in maintaining metabolic homeostasis and impacts various aspects of health [2]. Research shows that dysbiosis, or alterations in the gut microbiota, can contribute to the development of type 2 diabetes (T2DM) by affecting glucose metabolism, insulin sensitivity, and inflammation [3]. Additionally, the gut microbiota is linked to the emergence of obesity, metabolic syndrome, and the onset of T2DM through mechanisms such as impaired glucose tolerance and increased insulin resistance [3]. Recent meta-analyses support this by demonstrating that microbiota-derived interventions significantly improve serum levels of fasting insulin and hemoglobin A1c, both of which are key markers of disease progression in T2DM patients [4]. Various strategies have been explored to modulate gut microbiota, including the use of probiotics, prebiotics, and synbiotics. Probiotics are live microorganisms that provide health benefits when ingested, while prebiotics are non-digestible compounds that stimulate the growth of beneficial gut bacteria. Synbiotics, a combination of probiotics and prebiotics, work synergistically to enhance gut health [5]. Research into the role of probiotics and synbiotics in T2DM is rapidly expanding, emphasizing their positive effects on glycemic control and other metabolic parameters. However, the vital role of prebiotics as a nutritional substrate for these bacteria is often overlooked. Different types of prebiotics can directly impact diabetes by influencing blood sugar levels and indirectly by supporting the growth of beneficial bacteria. This study seeks to fill this critical gap by comprehensively analyzing the relationship between various prebiotics and T2DM. First, we will describe in detail different prebiotics, their suggested daily dosages, the foods that contain them, their direct effects on T2DM parameters, and the molecular mechanisms driving these beneficial effects, along with the resulting changes in gut microbiota. We will also critically evaluate the true efficacy of specific prebiotics in this context. Next, we will explore the mechanisms by which prebiotics improve glycemic indices, including their influence on key metabolites, anti-inflammatory effects, incretin secretion optimization, improvements in lipid profiles, and antioxidant properties. Through this holistic approach, this study aims to provide a deeper understanding of how prebiotics can be harnessed as a therapeutic strategy to combat T2DM.

## 2. Effects of Specific Prebiotics on Microbial Composition and T2DM

To be classified as a prebiotic, a food ingredient must meet specific criteria: it must resist gastric acidity, avoid hydrolysis by digestive enzymes, undergo fermentation by gastrointestinal microflora, and increase the abundance of health-promoting intestinal bacteria [6]. Prebiotics essentially serve as non-digestible food substrates that bypass human digestion, ultimately reaching the intestinal tract where they provide an energy source for colonic gut microbiota [7]. This relationship is symbiotic, as prebiotics promote the growth of beneficial gut microbiota, such as *Lactobacillus*, *Akkermansia*, *Bifidobacterium*, *Faecalibacterium*, and *Roseburia*, which help mitigate metabolic processes associated with T2DM [8].

Currently, carbohydrates are recognized as the most effective prebiotics [9], and they can be classified based on their molecular size or degree of polymerization [10]. However, a variety of food components, including non-digestible carbohydrates, specific proteins and peptides, and certain lipids, have also been identified as potential prebiotic ingredients [11]. Several prebiotics, such as inulins, fructooligosaccharides, galactooligosaccharides, resistant starches, pectic oligosaccharides, β-glucans, polyphenols, and *Dendrobium* spp., have been shown to exert a therapeutic effect on controlling glycemic indices in individuals with T2DM by optimizing gut microbial composition [12,13]. In the following sections, we will explore the functional properties of these prebiotics, emphasizing their impact on gut microbial composition and their influence on glycemic indices in individuals with T2DM.

### 2.1. Inulin

**Figure 1:**
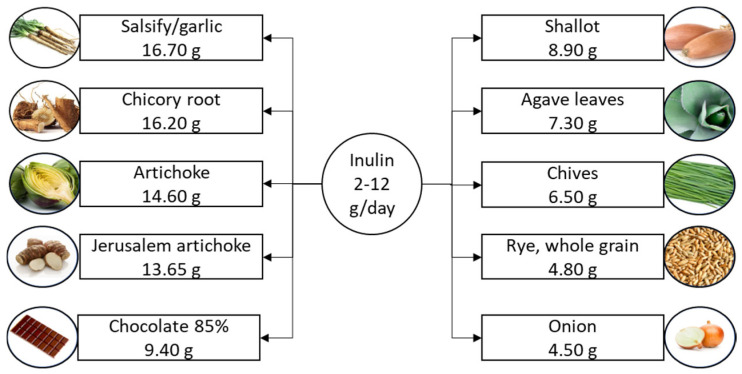
Suggested daily intake of inulin and the richest food sources. Inulin content is listed per 100 g of product.

Inulin, a water-soluble storage polysaccharide found in over 36,000 plant species, is part of the non-digestible carbohydrate group known as fructans. As a classified prebiotic, inulin occurs naturally in a variety of foods [14], as illustrated in Figure 1. The suggested daily intake ranges from 2 to 12 g [11].

Inulin exhibits significant prebiotic properties, with longer-chain inulin-type fructans demonstrating stronger effects on fermentation activity and the composition of bacterial communities [15]. When combined with fructooligosaccharides, it is regarded as a model prebiotic [16]. Inulin’s pharmacological properties make it a versatile ingredient across various food categories. It functions as a low-calorie sweetener and a non-digestible fiber, contributing to viscosity enhancement, gel formation, and improved sensory attributes in food products [17]. Notably, inulin enables the development of low-fat meat and poultry products with desirable textures and sensory qualities [18]. Its fat-replacing and texture-modifying properties make it applicable to a wide range of food items [19]. Inulin is used in various food products, including as a fat replacer in meat products, dairy items, sauces, and candies [20]. Its extensive use as a food additive in bread, bakery products, dairy, confectionery, and baby food underscores its significant role in the food industry [21].

Several studies suggest the potential benefits of inulin for managing T2DM. In one study involving 49 women with T2DM and a Body Mass Index (BMI) between 25 and 35 kg/m², it was found that taking 10 g of inulin daily for 2 months led to reductions in fasting plasma glucose and hemoglobin A1c (HgbA1c) levels, along with an increase in total antioxidant capacity [22]. Similarly, another study involving 52 women with T2DM showed that supplementation with 10 g of oligofructose-enriched inulin for 8 weeks also reduced fasting plasma glucose and HgbA1c levels [23]. Additionally, a double-blind crossover study found that taking 30 g of inulin daily for 2 weeks improved insulin sensitivity in individuals with prediabetes compared to a control group [24]. Moreover, supplementing with 15 g of inulin per day for 6 months resulted in a reduction in fasting insulin levels and an improvement in the homeostatic model assessment for insulin resistance (HOMA-IR) in patients with prediabetes [25]. However, a randomized, double-blind trial reported that consuming 10 g of inulin daily for 12 weeks did not significantly affect cholesterol, blood sugar, or HgbA1c levels in patients with T2DM [26]. Despite this, there is considerable evidence supporting the potential of inulin as a therapeutic option for individuals with T2DM.

Regarding the effects of inulin on gut microbiota, it has been observed that administering 15 g of inulin daily for 6 months to patients with prediabetes led to an increased relative abundance of Actinobacteria, Bifidobacteriales, *Bifidobacteriaceae*, *Lactobacillaceae*, *Bifidobacterium*, *Lactobacillus*, and *Anaerostipes* at both 3 and 6 months, along with a decrease in the relative abundance of *Alistipes* [25]. Similarly, an increase in the abundance of Actinobacteria and *Bifidobacterium* was observed following the administration of 5 or 7.5 g of agave inulin per day for 21 days in healthy adults, with a reduction in *Desulfovibrio* abundance [27]. In another study, supplementation with 12 g of chicory-derived Orafti inulin daily for 4 weeks resulted in an increase in *Bifidobacterium* and *Anaerostipes* spp., as well as a decrease in *Bilophila* [28]. Similar findings were reported by Baxter et al. [29], who documented an increase in *Bifidobacterium*, *Anaerostipes hadrus*, and *Eubacterium rectale* in healthy adults after 2 weeks of inulin supplementation. Furthermore, the administration of 16 g per day of inulin-type fructans for 3 months in obese women increased the abundance of Bifidobacterium and *Faecalibacterium prausnitzii* while decreasing *Bacteroides intestinalis*, *Bacteroides vulgatus*, and *Propionibacterium* [30]. Importantly, this bifidogenic effect is consistently associated with higher fecal short-chain fatty acid (SCFA) concentrations, which may contribute to the beneficial effects on T2DM in affected populations [31].

In murine models, many of the molecular benefits of inulin supplementation on glycemic control have been well described, with some translation to human studies as well [32]. For example, in T2DM animal models, inulin supplementation has been shown to induce significant anti-inflammatory effects through the modulation of enteric glial cells, driven by positive changes in microbial composition, including an improved Firmicutes/Bacteroidetes ratio [33]. Specifically, increased butyrate levels were found to inhibit the NF-κB pathway, reducing the expression of pro-inflammatory interleukins and tumor necrosis factor (TNF). Similar anti-inflammatory effects were observed in another study, where decreases in plasma LPS, IL-6, TNF, and IL-17a were positively associated with improvements in fasting blood glucose and gut microbial composition [34].

These findings have been translated to humans, demonstrating the overall anti-inflammatory benefits of inulin supplementation. In a study involving 60 individuals with diabetes, inulin supplementation led to decreased expression of TLR4, NF-κB, and IL-1 [35]. Notably, butyrate played a key role in driving these changes, with antioxidant properties observed alongside anti-inflammatory effects, including improvements in antioxidant capacity and superoxide dismutase activity [35]. Inulin has also been shown to improve markers of insulin resistance, including the expression of specific genes and insulin receptor substrates [36,37]. In a longitudinal study of 67 patients with T2DM, a 10 g daily supplementation over two months resulted in decreased methylation of the INS gene [36]. Interestingly, INS gene methylation has been reported in the insulin promoter of pancreatic islet cells, providing further insight into the role of epigenetic modifications following inulin intervention in humans [38]. Although the epigenetic modification of the IRS1 gene was less pronounced compared to INS, it still showed a trend toward decreased methylation in association with improved metabolic parameters [36]. Additionally, in murine models, the IRS-1 and MAPK signaling pathways were affected following 8 weeks of chicory inulin supplementation [37]. The study demonstrated upregulation of IRS activity and inhibition of the mitogen-activated protein kinase (MAPK) pathway, which is known to contribute significantly to T2DM pathogenesis and complications like diabetic kidney disease due to its cytotoxic effects [39]. Lastly, in a study of 60 patients with T2DM, inulin supplementation, in conjunction with butyrate, improved markers of glycemia, lipid profiles, and GLP-1 secretion. Overall, there is substantial evidence supporting the role of inulin in improving glycemic indices, mitigating molecular processes associated with T2DM pathogenesis, and enhancing gut microbial composition.

### 2.2. Resistant Starch

**Figure 2:**
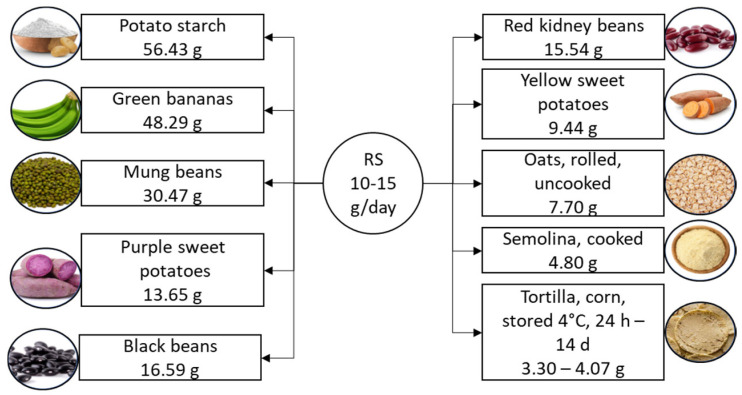
Suggested daily intake of resistant starch and the richest food sources. Resistant starch content is listed per 100 g of product. RS, resistant starch.

In the realm of carbohydrates, resistant starches possess unique properties, as they resist digestion by endogenous amylases in the small intestine, reaching the large intestine where they serve as nourishment for gut bacteria [40]. This distinctive characteristic classifies resistant starch as a dietary fiber, offering a range of potential health benefits [41]. Resistant starch is categorized into five types and naturally occurs in foods such as whole grains, legumes, cooked and cooled potatoes, rice, and unripe bananas [41,42,43], as illustrated in Figure 2.

Resistant starch can also be produced through various methods [41,44], and its properties can be further modified by processing techniques such as fermentation, extrusion, and chemical treatments [45,46,47]. These modifications can enhance its structure, fermentation properties, and resistance to digestion [45]. The suggested daily intake of resistant starch is 10–15 g [11]. Resistant starch is a versatile ingredient gaining popularity in the food industry due to its unique properties. It has a low caloric value, making it ideal for adding fiber and bulking up products like cereals, snacks, pasta, and baked goods without significantly increasing the calorie content [48]. In addition to its nutritional benefits, resistant starch improves the texture, consistency, and stability of food products. Various natural sources, such as legumes and cereals, offer resistant starch, increasing its appeal to manufacturers [49,50]. Various types of resistant starch have been identified, including physically inaccessible starch (resistant starch 1), enzyme-resistant starch (resistant starch 2), retrograded starch (resistant starch 3), and chemically modified starch (resistant starch 4) [51]. These types of resistant starch impact glucose responses in humans differently [52]. A 2023 meta-analysis reported a reduction in postprandial blood glucose following supplementation with resistant starch types 1 and 2, and a decrease in postprandial insulin response with resistant starch type 2 (RS2) in patients with T2DM or prediabetes [53]. Additionally, Wang et al. [54] found in their meta-analysis a reduction in fasting insulin, homeostatic model assessment of beta cell function (HOMA-B), HgbA1c, and an increase in homeostatic model assessment of insulin sensitivity (HOMA-S) in both healthy individuals and patients with diabetes following resistant starch supplementation. Fasting glucose levels were also reduced in patients with diabetes.

A 2020 meta-analysis further indicated a decrease in fasting plasma glucose following resistant starch supplementation, with greater improvements observed with intakes exceeding 28 g per day or interventions lasting longer than 8 weeks [55]. This meta-analysis also showed a reduction in HOMA-IR following resistant starch supplementation [55]. The mechanisms by which resistant starch improves glucose control involve its antioxidant and anti-inflammatory properties. Another meta-analysis, which included 16 trials and 706 patients with T2DM, demonstrated an increase in total antioxidant capacity and a decrease in inflammatory markers, such as CRP, IL-6, and TNF concentrations [56]. These findings consistently highlight the benefits of resistant starch in managing glucose levels and reducing inflammation in individuals with T2DM. In addition to these meta-analyses, individual clinical trials provide strong evidence supporting the incorporation of resistant starches into the diets of patients with T2DM, offering more detailed insights into the metabolic improvements associated with their consumption [57,58]. For example, supplementation with 10 g/day of RS2 for 8 weeks in 60 women with T2DM resulted in decreased HgbA1c, lower triglycerides, and reduced pro-inflammatory TNF-α, while also increasing serum HDL levels [58]. Interestingly, RS2 supplementation in another study with T2DM patients showed beneficial effects on postprandial GLP-1, leading to improved insulin responses after meals [57].

Research on the effects of resistant starch on gut microbiota composition has demonstrated that a diet rich in resistant starch, including RS2, led to an increase in the proportion of Firmicutes to Bacteroidetes, as well as a rise in *Faecalibacterium prausnitzii*, *Prevotellaceae*, *Ruminococcus*, *Eubacterium rectale*, *Roseburia faecis*, and *Akkermansia muciniphila* in individuals with low insulin sensitivity [59]. Conversely, in a study involving children who received 8.5 g of RS2 per day for 4 weeks, an increase in Actinobacteria and a decrease in Firmicutes were observed [60]. At the genus level, there was an increase in *Lactobacillus* and a decrease in *Roseburia*, *Blautia*, and *Lachnospiraceae incertae sedis* [60]. Different types of resistant starches elicit varied responses in gut microbiota composition, with RS4 potentially promoting Bacteroidetes, while RS2 favors Firmicutes [61,62]. Martinez et al. [62] also investigated gut microbiota changes following the administration of different types of resistant starch. They reported an increase in the Actinobacteria and Bacteroidetes phyla, along with an increase in *Bifidobacterium adolescentis* and *Parabacteroides distasonis* species, and a decrease in Firmicutes in participants who consumed 100 g of crackers containing RS4 compared to those who consumed crackers with RS2. Conversely, individuals who consumed crackers with RS2 exhibited an increase in *Ruminococcus bromii* and *Eubacterium rectale* species. Both types of resistant starch were associated with an increase in *Clostridium clostridioforme* proportions [62].

### 2.3. Fructooligosaccharides

**Figure 3:**
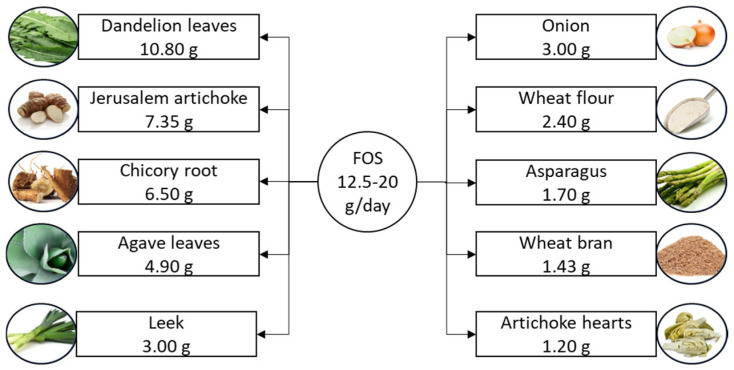
Suggested daily intake of fructooligosaccharides and the richest food sources. Fructooligosaccharide content is listed per 100 g of product. FOS, fructooligosaccharide.

Fructooligosaccharides are a subclass of oligosaccharides known for their well-documented prebiotic effects [63]. These short-chain carbohydrates typically range from trisaccharides to decasaccharides and are characterized by a terminal sucrose unit [64]. Due to their prebiotic properties, fructooligosaccharides are widely used in the production of functional and low-calorie food products [65]. They serve as sweetening agents and biopreservatives, making them valuable components in food formulation [66]. The suggested daily intake of fructooligosaccharides is 12.5–20 g [11], and they naturally occur in various plant sources, including onions, chicory, garlic, bananas, and artichokes [14,67], as illustrated in Figure 3.

The effects of fructooligosaccharide (FOS) supplementation on patients with T2DM have yielded inconsistent findings. One study reported that administering 8 g per day of FOS for 14 days led to a reduction in serum glucose concentrations in patients with poorly controlled T2DM [68]. However, another study by Alles et al. [69] found that administering 15 g per day of FOS for 20 days had no significant impact on blood glucose or lipid profiles in patients with T2DM. Similar results were observed in another study, where 20 g of FOS was administered for 4 weeks, with no significant effects on glucose or lipid parameters [70]. Interestingly, when combined with other prebiotics, fructooligosaccharides show improved metabolic control [71]. For example, when paired with polyphenols, the combined prebiotic effect improved pancreatic β-cell function, reduced hepatic insulin resistance, and decreased LDL cholesterol levels [71]. Significant changes in gut microbiota were also noted, including a fourfold increase in *Bifidobacterium* spp. and a twofold increase in *Eubacterium* spp. [71]. Supporting these findings, a 2022 meta-analysis reported an increase in *Bifidobacterium* spp. concentrations following FOS administration (7.5–15 g per day for over 4 weeks), with no significant changes in *Lactobacillus* spp. or *Enterobacteriaceae* [72]. FOS treatment also affected the gut microbiota differently across age groups, with a significant decrease in *Odoribacter* in adults and older adults, as well as reductions in *Bilophila* and *Lachnospiraceae* across all age groups, and a decrease in *Oscillospira* in young adults and adults [73]. Researchers identified potential compensatory taxa, including *Bacteroides*, *Megamonas*, *Collinsella*, and *Ruminococcus*, which showed non-significant increases in abundance [73].

On a molecular level, FOSs have demonstrated positive effects on the secretion of incretin hormones, such as GLP-1, in murine models—commonly used in T2DM treatment. FOSs alleviated apoptosis of intestinal L-cells while enhancing GLP-1 secretion in a T2DM model [74]. Similarly, FOS-containing biscuits have been shown to increase GLP-1 concentrations and lower blood sugar in vitro [75]. However, in humans, acute intake of FOS did not produce similar results, with no significant changes in intestinal hormone levels or satiety [76]. Likewise, FOS-rich syrup did not lead to significant changes in postprandial ghrelin or GLP-1 levels [77]. Given the mixed results and lack of strong evidence in human studies, fructooligosaccharides are not as strongly supported for improving glycemic indices compared to other prebiotic substrates discussed in this review.

### 2.4. Galactooligosaccharides

**Figure 4:**
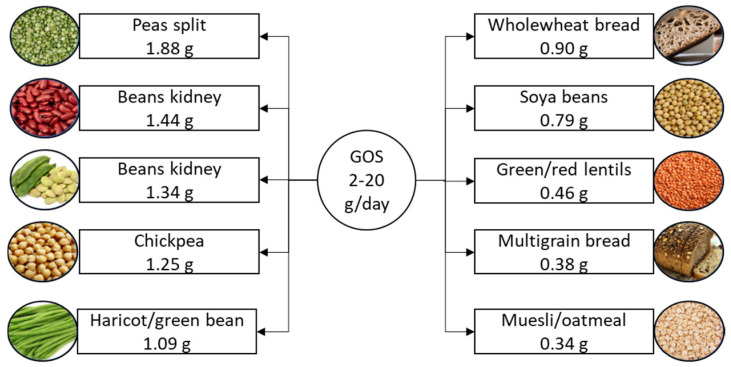
Suggested daily intake of galactooligosaccharides and the richest food sources. Galactooligosaccharide content is listed per 100 g of product. GOS, galactooligosaccharide.

Galactooligosaccharide, a naturally occurring functional oligosaccharide and key active component in milk, is a widely used prebiotic. It exists in two subtypes, α-galactooligosaccharide and β-galactooligosaccharide, which are differentiated by their specific galactosidic linkages [78]. Galactooligosaccharides are gaining attention in food production due to their dual functionality. These prebiotic carbohydrates not only improve the sensory qualities of processed foods, such as taste, texture, and foam stability [79], but also promote gut health by selectively stimulating the growth of beneficial bacteria, including bifidobacteria and lactobacilli [80]. For example, ice cream and yogurt can be enriched with galactooligosaccharides to provide digestive benefits [80]. Their low-calorie content, bulking capacity, and stability in acidic environments make galactooligosaccharides ideal for use in various processed food applications, including beverages, fermented milk products, confectionery, and baby food [81]. The suggested daily intake of galactooligosaccharides is 2–20 g [11], with the richest dietary sources shown in Figure 4.

Research on the effects of galactooligosaccharides (GOSs) on fasting insulin and blood glucose levels has yielded mixed results, similar to findings with fructooligosaccharides. In one study involving overweight individuals, a significant decrease in fasting insulin was observed after 84 days of supplementation with 5.5 g of GOSs daily [82]. Additional metabolic improvements noted in this study included reduced total cholesterol, triglycerides, and inflammatory markers such as CRP [82]. Conversely, another study on patients with T2DM who received the same dosage over the same duration did not observe significant changes in fasting blood glucose, HgbA1c, or fasting insulin levels [83]. However, this study did find that *Veillonellaceae* was inversely correlated with glucose response and inflammatory markers, such as IL-6, in patients with well-controlled T2DM [83]. Similarly, a study conducted in the Netherlands on overweight or obese individuals who consumed 15 g of GOSs daily for 84 days did not report significant changes in fasting blood glucose, fasting insulin, gut hormones, incretins, or markers of inflammation [84].

While the findings on fasting insulin and blood glucose are inconsistent, studies examining the impact of GOSs on gut microbiota have shown more consistent results. For example, a study on laboratory mice demonstrated an increase in *Bifidobacterium* levels following GOS supplementation [85]. In humans, GOS supplementation similarly increased *Bifidobacteriaceae*, although no improvement in glucose tolerance was observed in the short term [86]. Another 12-week study found that GOS supplementation selectively increased fecal *Bifidobacterium* fivefold, but did not significantly affect insulin sensitivity in 44 overweight/obese individuals with prediabetes. Interestingly, in a recent study involving 53 prediabetic individuals, a 12-week supplementation of both GOSs and Bifidobacterium breve resulted in significant reductions in HgbA1c and fasting blood glucose compared to placebo groups [87].

In addition to increasing Bifidobacteria, GOS supplementation has been associated with other beneficial changes in gut microbial composition. For example, in a study on patients with ulcerative colitis, daily administration of 2.8 g of GOSs for 6 weeks led to a reduction in Bacteroidetes levels and an increase in the abundance of *Bifidobacterium* and *Christensenellaceae* [88]. Marzorati et al. [89] reported increases in several beneficial bacterial species, including *Bifidobacterium longum*, *Bifidobacterium adolescentis*, *Lactobacillaceae*, and *Ruminococcus torques*, as well as a decrease in Clostridiales, *Erysipelotrichaceae*, *Odoribacteraceae*, and *Oscillospiraceae* following GOS supplementation. Overall, while the efficacy of GOSs in improving glycemic indices in T2DM remains inconclusive, their positive effects on gut microbiota are well documented. The data suggest that GOSs, when used in conjunction with probiotics or other beneficial agents, may enhance their efficacy, though supplementation alone may not lead to significant changes in glycemic outcomes.

### 2.5. Pectic Oligosaccharides

Pectins, essential polysaccharides found in the cell walls of higher plants, play a crucial role in maintaining the rigidity and structure of plant tissues [90]. The pectin content in fruits and vegetables ranges from 0.1% to 2.5%, and a daily intake of 30 g is suggested for significant health benefits, such as reducing postprandial glycemic responses, maintaining normal cholesterol levels, and increasing satiety, which may lead to reduced caloric intake [91]. Pectin is present in varying amounts across different sources, such as 34.4% in olive pomace, 30% in citrus waste, 27–34% in onion skin, 20.9% in apple pulp, 16.31% in soy hull, 16.2% in sugar beet pulp, and 15% in potato pulp [92]. Pectins can also serve as a source for pectic oligosaccharides (POSs), which exhibit structural differences depending on their source [93]. The suggested intake of POSs is 10–20 g per day, providing potential prebiotic benefits and supporting intestinal health [94].

Several studies have highlighted the antidiabetic properties of pectin. For instance, pectin derived from red chili fruit waste has been shown to significantly improve insulin sensitivity and lower blood glucose levels in diabetes models [95]. Additionally, the addition of 16 g of guar and 10 g of pectin to a meal containing 106 g of carbohydrates significantly reduced postprandial glucose and insulin levels in both insulin-dependent and non-insulin-dependent diabetics [96]. Similarly, a reduction in postprandial glucose was observed in healthy adults aged 19–33 years who consumed 10 g of pectin with a carbohydrate-containing meal [97]. Furthermore, a study involving 43 volunteers with T2DM who consumed 30 g per day of yellow passion fruit peel flour for two months demonstrated significant reductions in fasting blood glucose and HgbA1c levels post-supplementation. The HOMA-IR index decreased, indicating reduced insulin resistance, although no significant changes in insulin levels were observed in women. Instead, the HOMA-beta index increased significantly [98].

Pectin exerts its antidiabetic effects through multiple mechanisms. It forms gels in the gastrointestinal tract, slowing gastric emptying and reducing glucose absorption, thereby helping regulate blood glucose levels and increasing satiety, which contributes to lower caloric intake [99]. Additionally, pectin positively influences lipid metabolism, reducing cholesterol levels and lowering the risk of diabetes and cardiovascular diseases [100]. POSs derived from hawthorn have been shown to lower serum levels of total cholesterol and triglycerides and inhibit body fat accumulation [101]. Moreover, pectin has anti-inflammatory effects that may help mitigate the risk of insulin resistance by reducing inflammation markers and improving overall metabolic health [102].

The ability of pectin to modulate gut microbiota is another key factor, as it promotes the growth of beneficial bacteria, which can improve metabolic health and insulin sensitivity [103]. Pectin undergoes slow fermentation and exhibits prebiotic effects by producing short-chain fatty acids (SCFAs). Pectic oligosaccharides (POSs) have demonstrated bifidogenic potential and offer various health benefits, including anti-obesity, anticancer, and antioxidant properties [104]. POSs represent a new class of prebiotics that generate SCFAs through fermentation by gut microbiota. A study showed that POSs from sugar beet have the highest bifidogenic effect and the highest concentration of SCFAs, while POSs from citrus peel increased the Lactobacillus population [105]. Recent findings also indicated that SCFA concentrations were higher with POS supplementation compared to fructooligosaccharides (FOSs) [106].

Moreover, POSs from highly methylated citrus pectin and low-methylated apple pectin were shown to mitigate the toxicity of Shiga-like toxins from *Escherichia coli* O157 [107], while POSs from carrots blocked the adhesion of *Escherichia coli* to uroepithelial cells [108]. Another in vitro study found that *Bifidobacterium angulatum*, *Bifidobacterium infantis*, and *Bifidobacterium adolescentis* were able to utilize POSs, particularly low-methylated substrates [109]. Increased numbers of Bifidobacteria and lactobacilli have been observed with POSs derived from bergamot peel [110], sugar beet, and Valencia oranges [93], as well as from apple pectin [93] and a mixture of POSs from apple pulp [111]. POSs from bergamot peel also led to a decrease in Clostridium populations [110]. POSs from sugar beet and Valencia oranges promoted the production of acetate, butyrate, and propionate [93], while POSs from apple pectin increased concentrations of acetic acid, lactic acid, and propionic acid and reduced Bacteroides and Clostridia populations [112]. Similarly, POSs from apple pulp decreased Bacteroides and Clostridia populations [111], and POSs from orange peel increased numbers of *Bifidobacterium* and *Eubacterium rectale*, correlating with higher butyrate concentrations [113]. In an infant study, Fanaro et al. [114] reported a significant increase in bifidobacteria and lactobacilli in infants fed with formulas enriched with POSs. Similarly, Magne et al. [115] observed an increase in bifidobacteria and a decrease in *Bacteroides* and *Clostridium coccoides* in a group receiving a GOS/FOS/POS mixture, compared to a mixture containing only GOS/FOS. Therefore, compared to other oligosaccharides such as FOS and galactooligosaccharides (GOSs), research to date suggests that POSs may have stronger antidiabetic properties, as discussed in this review.

### 2.6. Polyphenols

Rich in essential nutrients, plant-based foods contain a variety of bioactive compounds, including phenols, carotenoids, and alkaloids, that offer beneficial effects on the body [116]. Phenolic compounds, which are biologically active secondary metabolites derived from plants, are abundant in vegetables, fruits, whole grains, and other plant sources. These compounds have garnered attention due to their anti-inflammatory, antioxidant, and metabolic-regulating properties [117]. Found in all plant families, phenols can reduce the risk of T2DM, and diversifying plant-based foods in the diet increases the intake of these valuable compounds. However, the concentration and composition of phenolic compounds can vary among different plant sources, and their bioavailability and effects are influenced by harvesting and processing conditions [118]. In Europe, the daily intake of phenolic compounds, mainly flavonoids and phenolic acids from fruits, chocolate, and vegetable juices, ranges from 167 to 564 mg, and this can increase to gram levels through nutraceuticals and fortified products [119]. Regular consumption of approximately 1–2 g of polyphenols daily is associated with the prevention of chronic diseases, and an intake of over 650 mg per day has been shown to significantly reduce the risk of death [120,121]. Recently, interest has grown in the potential of dietary phenolic compounds, such as flavonoids, coumarins, quinones, stilbenes, and curcuminoids, for managing diabetes. These compounds have been shown to enhance insulin secretion, regulate blood sugar levels, and may help prevent diabetes-related complications by influencing complex molecular processes [122]. For instance, bayberry extract has demonstrated hypoglycemic activity by enhancing glucose uptake in liver cells and boosting glutathione levels [123]. Polyphenols from young apples stimulate glucose absorption, improve mitochondrial function, and reduce oxidative stress [124]. Oregano, blackberry extract, white mulberry, and sprouted quinoa yogurt have also shown hypoglycemic, antioxidant, and anti-inflammatory effects, supporting diabetes management [125,126,127,128].

Polyphenols from various plants have shown significant potential in animal studies for diabetes management. For example, polyphenols from *Rumex dentatus* L., blueberry leaf, *Cochlospermum regium*, and *Vernonia amygdalina* have been effective in lowering blood glucose levels and improving insulin resistance, lipid profiles, and body weight in diabetic models [129,130,131,132,133,134]. Clinical trials have further explored polyphenols’ antidiabetic potential. A study involving 25 men at cardiovascular risk found that consuming 250 mL of Hibiscus sabdariffa extract with breakfast led to reductions in serum glucose, insulin, triglycerides, and C-reactive protein [135]. Another study with patients with T2DM who were supplemented with 160 mg of purified anthocyanins twice daily for 24 weeks showed reductions in LDL cholesterol, triglycerides, fasting plasma glucose, and insulin resistance, along with increases in HDL cholesterol and antioxidant capacity [136]. Furthermore, patients with T2DM who did not respond to oral antidiabetic medications showed significant reductions in fasting glucose, postprandial glucose, and HgbA1c after supplementing with 350 mg of bilberry extract every 8 h for 2 months, without adverse effects on liver or kidney function [137].

Bioactive phenolic compounds, including flavonoids and non-flavonoids, are partially absorbed in the stomach and small intestine, with the remainder reaching the large intestine. There, they are either utilized by the gut microbiota to exert prebiotic effects or transformed into active metabolites [138]. Approximately 90–95% of consumed phenols are not immediately absorbed but reach the large intestine, where they play a protective role in human health [139]. Various studies have shown that polyphenols increase the abundance of beneficial gut bacteria, such as *Lactobacillus* and *Bifidobacterium*, while modulating other microbiota, including Bacteroides, *Clostridium*, and *Faecalibacterium prausnitzii* [140,141,142,143,144,145,146]. These changes in gut microbiota may contribute to the antidiabetic, antioxidant, and anti-inflammatory effects of phenolic compounds, further supporting their role in metabolic health.

### 2.7. β-Glucans

β-glucans are soluble dietary fibers primarily found in oats and barley, recognized for their potential health benefits, particularly in managing diabetes [147]. These compounds are naturally present in plant cell walls, cereal seeds, and in certain fungi, yeasts, algae, and bacteria. They are highly concentrated in the cell walls of oat and barley endosperm, where they account for 75% of their content, and in bran, where they make up 10.4% [148]. Oats and barley have the highest β-glucan concentrations among cereals, with oats containing 3–8 g per 100 g of dry weight and a solubility of 82%, while barley contains 2–20 g with a solubility of 65%. In contrast, other cereals have significantly lower β-glucan levels [148].

Studies have shown that β-glucans can significantly improve glycemic control, increase insulin sensitivity, and lower cholesterol levels [147]. Their primary mechanism of action involves increasing intestinal viscosity, which slows carbohydrate absorption and, in turn, helps modulate postprandial glucose levels, preventing sharp spikes in blood glucose [72]. β-glucans are considered superior to other soluble fibers due to their ability to form highly viscous solutions at low concentrations (1%) and their stability across various pH levels [149]. Daily consumption of approximately 3 g of β-glucans has been shown to significantly improve glycemic control and reduce insulin resistance in individuals with type 2 diabetes [147,150]. Additionally, β-glucans have been associated with lower HgbA1c levels [150,151].

In addition to improving glycemic control, β-glucans positively affect lipid metabolism, contributing to reduced cardiovascular risk, particularly in diabetic individuals. They lower total and LDL cholesterol levels by binding to bile acids, promoting their excretion, and reducing cholesterol absorption in the intestines [147,148,150]. This lipid-lowering effect is linked to their ability to increase intestinal viscosity, which binds glucose, bile acids, and cholesterol, enhancing fecal excretion [152,153]. Moreover, β-glucans stimulate the production of short-chain fatty acids (SCFAs) through fermentation in the gut microbiota, which in turn regulate hormones like GLP-1 and PYY, leading to increased insulin secretion and enhanced satiety [154].

Although some conflicting information exists, β-glucan fermentation appears to promote healthful changes in gut microbiota [155]. Studies in diabetic animal models have shown an increase in *Akkermansia* following the administration of yeast β-glucan [156] and baker’s yeast β-glucan [157]. Oat β-glucan administration increased *Clostridium* and *Butyricoccus*, while reducing Bacteroides, *Lactobacillus*, *Oscillospira*, and *Ruminococcus* [158]. Additionally, studies on obese animals have reported an increase in *Bifidobacterium* [159,160], Bacteroides, *Lactobacillus*, and *Atopobium* following the administration of barley β-glucan [160], and an increase in *Akkermansia* after administering baker’s yeast β-glucan [157].

Various studies have examined the effects of cereal β-glucans, such as those found in barley and oats, on modulating the gut microbiota. In vitro fermentation of hull-less Tibetan barley increased beneficial bacteria such as *Pantoea*, *Megamonas*, Bifidobacteria, and *Prevotella*, as well as SCFA concentrations, including acetate, propionate, and butyrate [161]. In patients at risk for metabolic syndrome, consuming bread made with β-glucan-enriched barley flour increased populations of *Bifidobacterium* spp. and *Akkermansia municiphila* [162]. Animal studies have shown that low-molecular-weight β-glucans increased populations of *Bifidobacterium* and Bacteroides, while also boosting SCFA production, particularly acetate and butyrate [163]. Similarly, *Roseburia hominis* and *Ruminococcus* increased following the consumption of whole-grain barley pasta, while Fusobacteria and Firmicutes decreased [164]. Studies on oats have also demonstrated an increase in beneficial bacteria such as *Bifidobacterium* and *Lactobacillus* [165].

### 2.8. Dendrobium officinale

In recent years, *Dendrobium* spp. has gained attention for its significant prebiotic properties, particularly in the context of T2DM animal models. It has demonstrated notable benefits, such as reducing chronic inflammation, protecting against pancreatic β-cell dysfunction, stimulating GLP-1 secretion, and upregulating short-chain fatty acid (SCFA) concentrations by enhancing microbial diversity [166]. These effects lead to improvements in multiple metabolic parameters, including enhanced glucose tolerance, insulin resistance, and lipid profiles [167]. For example, in a prediabetic murine model, Dendrobium officinale was shown to mediate inflammation, repair islet damage, increase incretin hormone release, and improve insulin secretion by modulating inflammation—particularly by decreasing LPS-mediated TLR4 activation [168]. In this study, *Dendrobium officinale* supplementation increased the relative abundance of *Roseburia*, *Alloprevotella*, *Bacteroides*, *Bifidobacterium*, and *Lactobacillus*, along with upregulation of SCFA production and intestinal GPR43 expression, which contributed to the observed metabolic benefits [168]. Similar anti-inflammatory effects were seen in another study, where *Dendrobium officinale* enhanced metabolic parameters by increasing the abundance of *Allobaculum*, *Bifidobacterium*, and *Lactobacillus*, reducing inflammation via downregulation of the LPS/TLR-4 pathway, and strengthening the intestinal barrier [169].

Furthermore, *Dendrobium officinale* polysaccharides have been shown to promote glycemic control by decreasing hepatic gluconeogenesis enzymes and regulating signaling pathways such as AMP-PKA and Akt [168]. These alterations in hepatic metabolic processes have been further detailed in studies assessing lipid metabolism in T2DM murine models, where *Dendrobium* supplementation improved liver function through activation of the PPAR signaling pathway, leading to decreased serum lipid levels and improved insulin sensitivity [170]. Similar studies have reported decreased inflammation as a result of PPARγ activation after 4 weeks of *Dendrobium* treatment [170]. Although no randomized clinical trials have been conducted in patients with T2DM, promising evidence from animal models suggests that the prebiotic effects of *Dendrobium* spp. may warrant further investigation as an adjunct treatment for glycemic control.

## 3. Changes in Gut Microbiota in T2DM

The gut microbiota composition in patients with T2DM shows distinct alterations, providing important correlations between microbial taxa and related metabolites that either increase sensitivity to T2DM development or protect against it [171,172,173]. Specifically, patients with T2DM exhibit a reduction in beneficial butyrate-producing bacteria such as *Faecalibacterium prausnitzii*, *Roseburia*, and *Eubacterium rectale* [174,175]. Butyrate, a short-chain fatty acid (SCFA), plays a critical role in regulating appetite, body weight, and insulin resistance [176]. Additionally, reductions in *Clostridium*, another butyrate producer, have been documented in diabetic individuals [177,178]. Similarly, *Anaerostipes hadrus*, another beneficial bacteria, is less abundant in individuals with T2DM, and this reduction is associated with impaired glucose metabolism and increased insulin resistance [179]. Research also shows decreased levels of *Eubacterium rectale* in individuals with T2DM, linked to dysbiosis that worsens insulin resistance [180,181]. *Ruminococcus bromii*, important for glucose metabolism, is less abundant in diabetic patients, especially those with chronic pancreatitis [182]. Reduced levels of *Bifidobacterium adolescentis* and *Christensenellaceae* have been found in T2DM patients, with *Christensenellaceae* showing a negative correlation with HgbA1c levels [183,184,185]. At the genus level, *Clostridium* is depleted in individuals with prediabetes, and its reduction is negatively correlated with glucose levels, insulin resistance, and inflammation [186,187].

Certain bacteria within the *Actinobacteria phylum*, such as *Bifidobacterium*, are linked to a lower risk of developing T2DM, while *Anaerostipes* has been found to positively influence fasting blood glucose levels [188,189]. Other investigations have shown reduced levels of *Bacteroides intestinalis*, *Bacteroides*, and *Bacteroides vulgatus* in T2DM patients [190]. Early studies revealed significantly lower levels of Clostridia and Firmicutes in T2DM patients, with *Clostridium coccoides* and *Clostridium leptum* specifically reduced in newly diagnosed cases [191,192]. *Bifidobacterium adolescentis* and *Bifidobacterium angulatum* have been found to contribute to improved glycemic control, while increased *Oscillospiraceae* levels are associated with lower insulin resistance [3,183,193]. Higher levels of *Megamonas* have been linked to normal glucose tolerance compared to T2DM [194].

A systematic review of preclinical and clinical trials showed that *Bifidobacterium*, *Bacteroides*, *Faecalibacterium*, *Akkermansia*, and *Roseburia* were inversely associated with T2DM development, contributing to improved metabolism and gut health. In contrast, genera like *Ruminococcus*, *Fusobacterium*, and *Blautia* have been linked to an increased risk of T2DM [190]. T2DM patients exhibit an increase in potentially harmful bacteria such as *Escherichia, Prevotella,* and *Lactobacillus*, as reported by Ejtahed et al. [175] and Qin et al. [195]. Jiang et al. [178] also observed a significant rise in Proteobacteria in diabetic patients. Higher levels of *Collinsella*, particularly *Collinsella aerofaciens*, are frequently observed in T2DM patients [196]. Additionally, *Ruminococcus torques* is linked to insulin resistance and hyperglycemia, with levels decreasing after bariatric surgery and diabetes remission [197].

**Figure 5:**
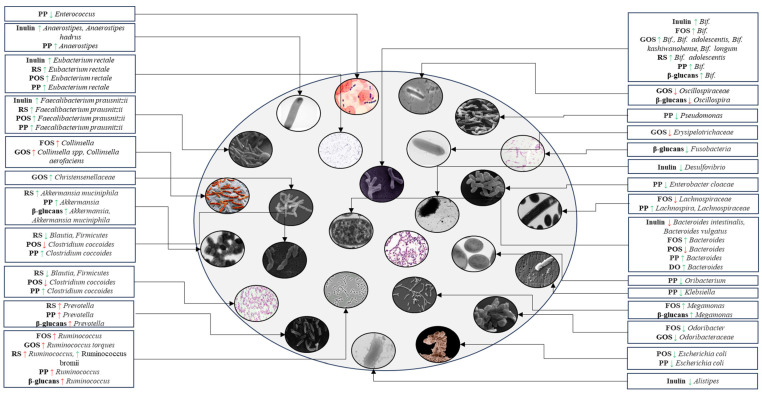
Schematic representation of gut microbiota composition in T2DM patients following administration of inulin, fructooligosaccharides, galactooligosaccharides, resistant starch, pectic oligosaccharides, polyphenols, β-glucans, and *Dendrobium officinale*. A green arrow indicates an increase or decrease in the abundance of bacteria that are typically less or more abundant in the gut microbiota of T2DM patients, contributing to a balanced microbial composition. A red arrow indicates potential increases in bacteria whose abundance is associated with T2DM patients or a decrease in beneficial bacteria that are typically less abundant in these patients. PP, polyphenol; RS, resistant starch; POS, pectic oligosaccharide; FOS, fructooligosaccharide; GOS, galactooligosaccharide; DO, *Dendrobium officinale*; *Bif*., *Bifidobacterium*; ↑, increase; ↓, decrease.

Certain *Bifidobacterium* species, including *Bifidobacterium adolescentis*, *Bifidobacterium bifidum*, *Bifidobacterium pseudocatenulatum*, *Bifidobacterium longum*, and *Bifidobacterium dentium*, show a negative association with T2DM, particularly in patients treated with metformin [184]. Butyrate-producing bacteria, such as *Clostridium leptum*, are negatively correlated with HgbA1c and fasting blood glucose levels [198]. Increased levels of Firmicutes are more prevalent in T2DM patients [199], while Lachnospira shows a negative association with fasting blood glucose in diabetic models [200]. Alistipes is linked to obesity and metabolic syndrome [201], and increased levels of Desulfovibrio and Odoribacter have been observed in T2DM [184,202]. Higher abundances of Erysipelotrichaceae are noted in cases of obesity and T2DM [203], and *Enterobacter cloacae* is associated with impaired glucose tolerance [204]. Furthermore, impaired immune responses in diabetes heighten the risk of infections by *Klebsiella pneumoniae* [205]. In metabolic syndrome, increased levels of Odoribacter have been observed [206], and a case study highlighted *Clostridium perfringens* causing a liver abscess in a diabetic patient, underscoring its opportunistic nature in immunocompromised individuals [207]. High-fat diets have also been linked to increased abundances of *Pseudomonas* in obese mice [208]. Changes in gut microbiota composition in response to prebiotics in T2DM are illustrated in Figure 5.

On the other hand, a balanced gut microbiota, known as eubiosis, is associated with improved glucose metabolism and increased insulin sensitivity in individuals with T2DM [209]. Additionally, eubiosis can help prevent complications related to T2DM, as a healthy gut microbiota may reduce the risk of diabetic complications such as retinopathy, nephropathy, and other related conditions [210]. Prebiotics selectively stimulate the growth of beneficial bacteria, enhancing microbial diversity. They do not promote harmful bacteria but can influence the growth of certain bacterial species in ways that may, at times, overlap with microbial changes observed in conditions like diabetes. As noted above, some bacterial types might be more abundant in individuals with diabetes. Therefore, administering prebiotics provides dual benefits for T2DM, promoting eubiosis while directly improving glycemic indices. The outcomes of these interventions are summarized in Table 1.

## 4. Mechanisms by Which Prebiotics Improve Glycemic Indices

**Figure 6:**
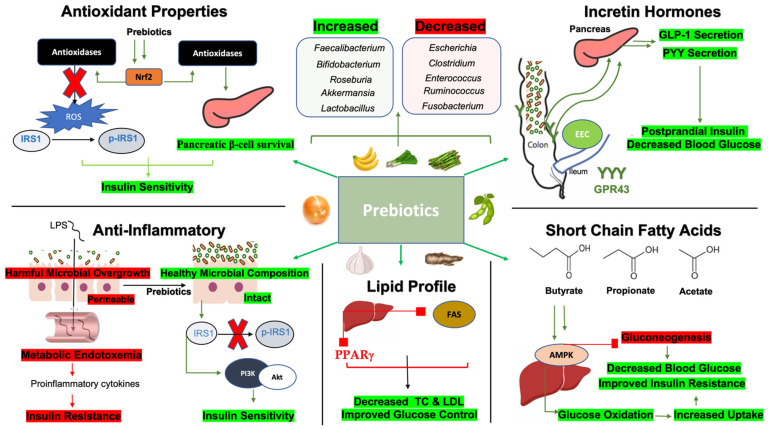
Microbiota-related mechanisms by which prebiotics improve glycemic indices. Short-chain fatty acids: Butyrate, propionate, and acetate enhance AMPK signaling in hepatic cells which inhibits gluconeogenesis through downstream signaling pathways. AMPK improves glucose oxidation for better uptake into tissue. These changes contribute to decreased blood glucose and improve insulin resistance. Anti-inflammatory: Inflammatory states in T2DM are associated with harmful bacterial overgrowth, leading to compromised intestinal barrier integrity and endotoxemia, where lipopolysaccharides (LPSs) leak into the bloodstream, causing metabolic inflammation. This process negatively impacts insulin resistance. Conversely, prebiotics enhance gut microbial composition, promoting better barrier integrity. These positive changes reduce the phosphorylation of insulin receptor substrate 1 (IRS1), improving downstream signaling and enhancing insulin sensitivity. Lipid profile: Prebiotics downregulate acetyl-CoA carboxylase (ACC) and fatty acid synthase (FASs), as well as peroxisome proliferator-activated receptor gamma (PPARγ). This promotes reduced total cholesterol and LDL while improving glucose control. Incretin Hormones: Prebiotics increase colonic microbial composition promoting the density of GPR43 receptors in the distal gastrointestinal tract. GPR43 activation leads to the induction of glucagon-like receptor 1 (GLP-1) and peptide YY (PYY) secretion, which improve postprandial insulin release and reduce postprandial glucose. Antioxidant properties: Prebiotics promote nuclear factor erythroid 2-related factor (Nrf2) transcription which induces the release of antioxidases. Antioxidases reduce reactive oxygen species (ROS) to reduce IRS1 phosphorylation and improve pancreatic β-cell survival, collectively improving insulin sensitivity. Abbreviations: AMPK, AMP-activated protein kinase; IRS1, insulin receptor substrate 1; p-IRS1, phosphorylated IRS1; PI3K, phosphoinositide 3 kinase; ACC, acetyl-CoA carboxylase; FAS, fatty acid synthase; PPARγ, peroxisome proliferator-activated receptor gamma; TC, total cholesterol; LDL, low-density lipoprotein; EECs, enteroendocrine cells; GLP-1, glucagon-like peptide 1; PYY, peptide YY; GPR43, G-coupled receptor 43; Nrf2, nuclear factor erythroid 2-related factor; ROS, reactive oxygen species.

In general, prebiotics improve blood glucose parameters through several mechanisms, including the fermentation of food products into bioactive short-chain fatty acids (SCFAs), reducing inflammation, increasing levels of blood glucose-lowering hormones, enhancing lipid metabolism, and boosting antioxidant enzyme activity [11]. The interplay of these molecular factors and pathways contributes to the observed benefits of prebiotic consumption in individuals with T2DM. These mechanisms are illustrated in Figure 6 and will be further explored in the following subsections.

### 4.1. Prebiotics, Short-Chain Fatty Acids (SCFAs), and Glycemic Indices

Prebiotics, primarily composed of soluble fibers, are fermented by gut microbiota through a series of reactions that produce short-chain fatty acids (SCFAs), namely acetate, propionate, and butyrate (2–4 carbon SCFAs) [212]. SCFAs, particularly butyrate, offer multiple benefits by serving as a key energy source for colonocytes [213]. Recent research has shown that regular fiber intake, a primary form of prebiotics, is associated with an approximately fourfold increase in SCFA concentrations in humans [214]. Notably, various prebiotic regimens have demonstrated consistent responses in microbiota composition and SCFA production [214]. SCFAs are crucial for improving insulin sensitivity by activating G-protein-coupled receptors (GPR41/GPR43) and initiating cellular energy homeostasis signaling pathways to enhance glucose uptake in peripheral tissues [215,216]. For example, a deficiency in prebiotic fibers in murine models correlates with reduced GPR41 and GPR43 signaling, leading to decreased cardiometabolic health [215]. Prebiotic supplementation has been shown to enhance hepatic AMP-activated protein kinase (AMPK) signaling, driven by increased SCFA-producing gut microbiota, thereby lowering insulin resistance [217]. AMPK activation improves insulin sensitivity by promoting glucose and fatty acid oxidation, enhancing uptake, inhibiting fat synthesis, and reducing energy-consuming processes like gluconeogenesis [218]. Propionate, in particular, suppresses hepatic gluconeogenesis via the GPR43/AMPK pathway, where activation of GPR43 leads to calcium-dependent AMPK activation in hepatocytes, thereby reducing gluconeogenesis [219]. In addition, prebiotics enhance anti-inflammatory effects, antioxidant properties, lipid metabolism, and incretin hormone release, in part by increasing SCFA production and promoting SCFA-producing bacteria. For example, dietary fiber intake has been shown to improve various parameters associated with T2DM, including inflammation, lipid profiles, and earlier satiety, through increased SCFA production [220]. These mechanisms will be further detailed in the following subsections.

### 4.2. Prebiotics, Anti-Inflammatory Properties, and Glycemic Indices

T2DM is characterized by low-grade inflammation, driven by the release of pro-inflammatory cytokines from adipose tissue, macrophage infiltration, and harmful gut microbial species and their metabolites [221]. This inflammation is often clinically associated with elevated levels of C-reactive protein (CRP). A meta-analysis of interventions involving prebiotics demonstrated a significant reduction in CRP levels, lower circulating tumor necrosis factor-alpha (TNF-α), and improvements in antioxidant enzyme activity in patients with T2DM [222]. Additionally, prebiotics such as resistant starches, resistant dextrin, and fructooligosaccharides have shown notable glycemic and anti-inflammatory benefits in another meta-analysis of 27 studies [223]. Specifically, 19 of these studies reported improvements in glycemic parameters, including HgbA1c, HOMA-IR scores, and blood glucose, with many also noting enhanced anti-inflammatory effects [223].

The contribution of gut microbiota to low-grade inflammation in T2DM is linked to reduced gut barrier integrity and the production of endotoxins, particularly lipopolysaccharides (LPSs), which leak into the bloodstream and promote metabolic endotoxemia [224]. Increased circulating levels of LPSs, pro-inflammatory cytokines, and markers of gut barrier permeability, such as zonulin (ZO-1), are associated with poor glycemic control and the pathogenesis of T2DM [225]. A LPS, a component of the outer membrane of Gram-negative bacteria, acts as a potent inflammatory stimulus by binding to its receptor, Toll-like receptor 4 (TLR-4), triggering the release of systemic pro-inflammatory cytokines [226]. These cytokines, particularly in adipose tissue and the liver, contribute to insulin resistance by interfering with insulin signaling pathways [227]. For instance, cytokines like interleukin-6 (IL-6) and TNF-α activate kinases that phosphorylate insulin receptor substrate 1 (IRS-1), impairing insulin signaling [228]. Additionally, IL-6 induces the expression of a suppressor of cytokine signaling 3 (SOCS3), which degrades IRS-1, further worsening insulin resistance [229]. Furthermore, interleukins activate nuclear factor kappa beta (NF-κB), stimulating cytokine release and creating a feedback loop that exacerbates glycemic dysregulation [230].

Prebiotics have been shown to counteract these inflammatory processes by exerting anti-inflammatory effects [169,231,232]. For example, polysaccharide supplementation has been shown to reduce LPS leakage and metabolic inflammation in T2DM [169]. Mechanistically, prebiotics upregulate tight junction proteins, improving gut barrier integrity and mitigating LPS- and NF-κB-mediated inflammatory damage and oxidative stress. Concurrently, a twofold increase in beneficial SCFA-producing *Bifidobacterium* and *Lactobacillus* populations was observed, along with inhibition of harmful Helicobacter species [169]. Further, studies have shown that SCFA treatment in LPS-stimulated inflammatory cells reduces TNF-α and interferon-gamma (IFN-γ) in both normoglycemic and poorly controlled T2DM individuals [232]. Isomaltodextrin, another potential prebiotic, has been found to suppress TNF-α and IL-6, modulating immune responses by inhibiting macrophage infiltration in adipose tissue and restoring IRS-1 expression [233]. It also enhanced concentrations of *Bacteroides*–*Prevotella* and improved microbial diversity, leading to better insulin sensitivity [233]. Similarly, resistant starch supplementation has been shown to restore IRS-1 expression and its downstream signaling targets, including phosphoinositide-3-kinase (PI3K) and Akt [234]. Overall, inflammation is a significant contributor to poor glycemic control, and prebiotics play a crucial role in mitigating inflammatory signaling pathways associated with T2DM onset.

### 4.3. Prebiotics and Incretin Hormones

Incretin hormones, such as glucagon-like peptide 1 (GLP-1) and gastric inhibitory polypeptide (GIP), are well-known enhancers of postprandial insulin secretion and are currently popular pharmaceutical agents for improving metabolic health. Interestingly, prebiotic intake can naturally stimulate the release of GLP-1, peptide YY (PYY), and GIP by altering gut microbiota composition and its metabolites [235]. For example, a 2-week administration of prebiotics significantly correlated with increased plasma GLP-1 and PYY concentrations (r = 0.85) and decreased postprandial plasma glucose levels (r = −0.73) after a standardized meal [236]. The enhancement of GLP-1 release through prebiotics can be partly attributed to increased SCFA concentrations [237]. SCFAs bind to GPR41 and GPR43 receptors on GLP-1-secreting L cells, promoting cytosolic calcium release via Gq signaling, which in turn stimulates GLP-1 secretion [238]. Prebiotics such as oligofructose and inulin also promote these effects by upregulating GPR43 receptor expression and increasing the number of GLP-1-secreting L cells in the colon [239,240,241]. Specifically, fructooligosaccharide consumption has been linked to a twofold increase in enteroendocrine L-cell density within the terminal ileum and colon [240,241], while inulin has been shown to upregulate colonic GPR43 expression, leading to enhanced PYY secretion [239]. Kombucha, a tea rich in polyphenols and prebiotics, has demonstrated similar beneficial effects on pancreatic islet β-cell function by promoting GLP-1 and PYY release through increased levels of SCFA-producing bacteria such as *Butyricoccus*, *Lactobacillus*, and *Lachnospiraceae* [242]. Other markers of glycemic control, including reduced LPS levels and improved gut barrier integrity, were also observed, along with reductions in harmful bacterial genera like *Desulfovibrio*, *Escherichia*, and *Shigella* after a 4-week intervention [242].

Overall, there is strong evidence supporting prebiotic-mediated increases in incretin hormone release, contributing to improved glycemic control.

### 4.4. Prebiotics, Lipid Metabolism, and Glycemic Indices

Another important outcome of prebiotic supplementation is the improvement of lipid profiles, which directly and indirectly enhances glycemic control [243]. Similar to other beneficial mechanisms discussed, prebiotics achieve better lipid regulation by promoting the growth of favorable, SCFA-producing gut microbiota [244]. For instance, propionate has been shown to inhibit hepatic cholesterol synthesis, reduce triglyceride formation, and lower very-low-density lipoprotein (VLDL) secretion by downregulating key enzymes involved in cholesterol production, such as acetyl-CoA carboxylase (ACC) and fatty acid synthase (FAS) [245]. Butyrate also promotes fatty acid oxidation, reducing lipid accumulation in the liver, muscle, and adipose tissue [246]. These improvements in glycemic control are largely due to decreased ectopic fat deposition, as the inability to suppress lipolysis is a significant contributor to insulin resistance in patients with T2DM [247]. Often, these metabolic benefits occur simultaneously, as prebiotic supplementation with β-glucan has been shown to reduce markers of insulin resistance (AMPK signaling) and fatty acid storage (peroxisome proliferator-activated receptor γ, or PPARγ) [248]. Clinically, these effects are reflected in studies showing that resistant dextrin supplementation for 8 weeks resulted in decreased fasting plasma glucose, HgbA1c, and LDL-c levels, while increasing HDL concentrations [249]. Thus, the role of prebiotics in controlling lipid parameters is crucial, as lipid metabolism is intricately connected with both the development and management of T2DM.

### 4.5. Prebiotics, Antioxidants, and Glycemic Indices

Oxidative stress, driven in part by the overgrowth of harmful microbial species, plays a significant role in the pathogenesis and progression of T2DM by disrupting insulin signaling pathways and promoting pancreatic β-cell dysfunction [250]. Specifically, pathways mediated by reactive oxygen species (ROS) trigger apoptotic signals in pancreatic islet cells, leading to cell damage and diminished functional capacity [251]. In states of oxidative stress, such as those seen in T2DM, insulin sensitivity is further impaired by the phosphorylation of IRS-1 [252]. Additionally, pro-oxidants damage endothelial cells, reducing the production of nitric oxide, which is essential for glucose delivery to cells through its vasodilatory effects [253]. Bacterial genera such as *Escherichia*, *Clostridium*, and *Enterococcus*, as well as an increased ratio of Proteobacteria and Firmicutes to Bacteroidetes, are associated with heightened oxidative stress in T2DM [254,255].

Prebiotics, particularly polyphenols, mitigate oxidative stress by increasing the relative abundance of beneficial bacteria with inherent antioxidant capacities, which are effective at scavenging ROS [256]. Polyphenols are metabolized by gut microbiota to produce phenolic acids, potent antioxidants known to enhance insulin signaling, protect pancreatic β-cells, and improve glucose homeostasis [256]. Mechanistically, phenolic acids have been shown to reduce HOMA-IR scores by 20% through the downregulation of NADPH oxidase and the upregulation of nuclear factor erythroid 2-related factor 2 (Nrf2), a potent stimulator of antioxidant enzymes [257]. The same study demonstrated enhanced downstream insulin signaling, which notably increased Akt phosphorylation, indicating improved insulin sensitivity [257]. Curcumin, another polyphenol, promotes the survival and function of islet cells while reducing apoptosis by upregulating antioxidant enzymes such as glutathione peroxidase and superoxide dismutase [258]. Additionally, polyphenols have been shown to reduce lipid peroxidation specifically in T2DM patients, optimizing lipid profiles by decreasing LDL and increasing HDL levels [259]. These improvements are linked to increased antioxidant defense mechanisms, including elevated total glutathione levels, which help prevent the progression of diabetic complications [259]. Thus, the antioxidant properties of prebiotics play a critical therapeutic role in managing oxidative stress and controlling glycemic indices in T2DM.

## 5. Conclusions

Inulin, fructooligosaccharides, galactooligosaccharides, resistant starch, pectic oligosaccharides, polyphenols, β-glucans, and *Dendrobium officinale* present a promising approach to diabetes management beyond their role as probiotic supports. Research suggests that inulin, resistant starches, POSs, polyphenols, and β-glucans offer substantial benefits as prebiotics for individuals with diabetes, though the evidence for fructooligosaccharides and galactooligosaccharides is less convincing. When administered in sufficient quantities over an extended period, these prebiotic dietary fibers can directly enhance glucose metabolism and insulin sensitivity, leading to improved glycemic control. Additionally, they indirectly influence diabetes management by modulating gut microbiota, promoting a healthier balance between beneficial and harmful bacteria. The diverse alterations in gut microbial composition observed with different prebiotics highlight the need for personalized approaches. Emerging polysaccharides and potential prebiotic agents, currently being studied in murine models, may offer additional benefits for human health in the future. *Dendrobium officinale* is one such prebiotic with promising potential to improve insulin sensitivity, reduce inflammation, suppress gluconeogenesis, and enhance glycemic control.

As research in this field continues to evolve, the use of these prebiotic agents as adjunct therapies for diabetes management holds significant therapeutic potential. Ultimately, tailoring prebiotic interventions to individual gut microbiota profiles and metabolic characteristics is a well-supported strategy to optimize their effectiveness in improving glycemic control and overall health outcomes.

## Figures and Tables

**Table 1:** Effects of prebiotics on glycemic indices in individuals with T2DM.

Prebiotic	Study Type/Study Duration/Prebiotic Dosage	Results/Implications	Reference
Inulin	RCT/2 months/10 g	9% decrease in fasting blood glucose 10.5% decrease in HgbA1c levels ~19% increase in total antioxidant capacity Insulin resistance markers unchanged in this study	[22]
	RCT/8 weeks/10 g	9.5% decrease in fasting blood glucose 8.4% decrease in HgbA1c 8% decrease in IL-6 and ~20% decrease in TNFα 31.7% decrease in CRP	[23]
	Randomized Crossover Trial/2 weeks/30 g	Significant increase in incremental postprandial insulin release at 30 min and 60 min Significant reduction in insulin resistance as measured by HOMA-IR score	[24]
	Prospective Single-Arm Study/6 months/15 g	Decreased fasting glucose, 2 h post-OGTT insulin Improved HOMA-IR score Increased relative abundance of *Bifidobacterium* and *Lactobacillus* Decreased *Alistipes*	[25]
	RCT/12 weeks/10 g	No significant effects on cholesterol, blood sugar, or HgbA1c	[26]
	RCT/6 weeks/16 g	Significant increase in *Bifidobacterium* in T2DM patients Significantly higher fecal concentrations of total SCFA	[31]
	RCT/45 days/10 g	Decreased relative expression of TLR4, NF-κB1, Caspase-1, NLRP3, IL-1β, and IL-18 Improved total antioxidant capacity Increased superoxide dismutase and catalase enzymatic activity	[35]
	Longitudinal/2 months/10 g	*INS* gene unmethylation allows for improved insulin sensitivity and metabolic parameters *IRS1* gene methylation observed through findings is not significant	[36]
	RCT/45 days	Improved glycemic indices, lipid profile, and GLP-1 secretion	[211]
Resistant Starches	Meta-Analysis of 36 RCTs	Resistant starch type 2 improved acute postprandial insulin response Resistant starch types 1 and 2 improved postprandial glucose Resistant starch type 2 improved fasting glucose	[53]
	Meta-Analysis of 13 Case–Control Studies	Resistant starch reduced fasting insulin and fasting glucose while increasing insulin sensitivity Metabolic parameters including LDL concentration and HgbA1c were improved	[54]
	Meta-Analysis of 19 RCTs	Effects of fasting insulin and glucose tolerance test were not significant Effect size on improving fasting glucose was larger when resistant starch dose was greater than 28 g/day and intervention period was greater than 8 weeks	[55]
	Meta-Analysis of 16 RCTs	Improved total antioxidant capacity Reduced CRP concentration in T2DM patients Reduced IL-6 and TNF concentrations	[56]
	RCT/8 weeks/10 g	Resistant starch type 2 decreased Hgb A1c by 3%, TNF by 19%, and TG by 15% Increased HFL by 25% Changes in fasting blood glucose, CRP not significant in this study	[58]
	RCT/12 weeks/40 g	Resistant starch type 2 significantly lowered postprandial glucose Postprandial GLP1 was higher indicating beneficial effects on meal handling	[57]
Fructo- oligosaccharides	Randomized Crossover Study/20 days/15 g	FOSs did not significantly affect fasting blood glucose concentrations, serum total cholesterol, serum TG, serum free fatty acids, or serum acetate	[69]
	Double-Blind Crossover Study/4 weeks/20 g	FOSs had no effect on plasma glucose, insulin concentrations, or basal hepatic glucose production No effects were observed on glycated hemoglobin	[70]
	Randomized Crossover trial/8 weeks/Polyphenol + 8 g FOS	FOSs reduced hepatic insulin resistance Adding FOSs to polyphenols improved β-cell function Increased *Eubacterium* and *Bifidobacterium* Decreased *Ruminococcus gnavus*, a species correlated with increased hepatic insulin resistance in this study No effects were observed on plasma cholesterol or LDL	[71]
	Crossover RCT/Short-Term Intake (2 h)/20 g	Increased gastric emptying in the short term Reduction in small intestinal transit No changes in incretin hormones or subjective feelings of hunger or satiety	[76]
	RCT/180 min	FOS-containing yacon syrup had no effect on GLP-1 levels or subjective appetite sensation	[77]
Galacto- oligosaccharides	RCT/12 weeks	Increased concentrations of fecal *Bifidobacterium* spp. Decreased fecal calprotectin, plasma CRP, and serum total cholesterol Decreased serum insulin was noted	[82]
	RCT/12 weeks/5.5 g	No significant effects on clinical outcomes including glucose tolerance, intestinal permeability, and gut microbiota Changes in *Veillonellaceae, however,* correlated inversely with IL-6 and glucose response	[83]
	RCT/12 months/15 g	Increased abundance of *Bifidobacterium* spp. No differences in fecal SCFA concentrations No significant changes in incretins, LPSs, or other markers of inflammation No significant changes in insulin sensitivity	[84]
	RCT/4 weeks/10 g	No improvement in glucose tolerance during study period Marked restoration of *Bifidobacterium* spp. No significant effects on LPS-binding protein	[86]
Pectic Oligosaccharides	Clinical Trial/120 min/10 g	Markedly decreased postprandial blood glucose and significantly lowered insulin levels in non-insulin-dependent diabetes In insulin-dependent diabetics, similar results were shown in postprandial glucose	[96]
	Clinical Trial/45 min/10 g	Decreased postprandial glucose and insulin levels	[97]
	RCT/2 weeks/30 g	Significantly reduced fasting blood glucose values, and HgbA1c and HOMA-IR values were observed	[98]
	Randomized Crossover/180 min	Significant reduction in postprandial blood glucose and insulin responses throughout 180 min Glucose was lowered by 13.2%	[99]
Polyphenols	N/A: In Vitro Analysis	Polyphenolic extracts and digests from oregano exhibited cellular antioxidant capacity These extracts promoted hypoglycemic and hypolipidemic properties	[125]
	N/A: In Vitro Analysis	Significantly increased glucose consumption and glycogen content in hepatic cells Attenuated ROS overproduction and glutathione depletion in hepatic cells	[126]
	N/A: In Vitro Analysis	Upregulated GLP-1 release in dose-dependent manner Proglucagon, its precursor, and mRNA expression was increased 2.68-fold	[128]
	RCT/1–2 h/250 milliliters	Reduction in serum glucose, plasma insulin, serum TG, and CRP levels Significant improvement in antioxidant response	[135]
	RCT/24 weeks	Lowered fasting plasma glucose by 8.5% and improved HOMA-IR score by 13% β-hydroxybutyrate was elevated by 42.4% Significantly decreased serum LDL by 8% and TG by 23%, while increasing HDL by 19%	[136]
	RCT/2 months/350 mg every 8 h	Lowered fasting blood glucose, 2 h postprandial glucose and HgbA1c No significant effect on liver or kidney function	[137]
β-glucans	Controlled Trial/6 months/7 g	Reduction in HgbA1c by 0.5 points Postprandial and plasma glucose was decreased No significant change in body weight or plasma lipids	[147]
	RCT/90 min/4 g	GLP-1 was significantly reduced at 90 min Blood glucose was reduced at 30 min Plasma insulin was reduced at 30 and 60 min	[154]
	RCT/4 weeks/6 g	Increased SCFA concentrations with 43% increase in propionic acid Higher abundances of *Bifidobacterium* and *Akkermansia* in metabolic-responsive individuals	[162]

RCT, randomized control trial; HgbA1c, hemoglobin A1c; IL, interleukin, TNFα, tumor necrosis factor-alpha; CRP, C-reactive protein; HOMA-IR, homeostatic model assessment for insulin resistance; OGTT, oral glucose tolerance test; T2DM, type 2 diabetes mellitus; SCFAs, short-chain fatty acids; TLR4, Toll-like receptor 4; NF-κB, nuclear factor kappa beta; NLRP3, nucleotide-binding domain leucine-rich-repeat-containing protein 3; IRS1, insulin receptor substrate; GLP-1, glucagon-related peptide 1; LDL, low-density lipoprotein; TGs, triglycerides; FOSs, fructooligosaccharides; LPSs, lipopolysaccharides; ROS, reactive oxygen species.
